# Blood Levels of Macrophage Migration Inhibitory Factor after Successful Resuscitation from Cardiac Arrest

**DOI:** 10.1371/journal.pone.0033512

**Published:** 2012-04-10

**Authors:** Christian Stoppe, Michael Fries, Rolf Rossaint, Gerrit Grieb, Mark Coburn, David Simons, David Brücken, Jürgen Bernhagen, Norbert Pallua, Steffen Rex

**Affiliations:** 1 Department of Anaesthesiology, University Hospital of the RWTH Aachen, Aachen, Germany; 2 Institute of Biochemistry and Molecular Cell Biology, University Hospital of the RWTH Aachen, Aachen, Germany; 3 Department of Intensive Care, University Hospital of the RWTH Aachen, Aachen, Germany; 4 Department of Plastic Surgery, Hand Surgery, Burn Unit, University Hospital of the RWTH Aachen, Aachen, Germany; 5 Department of Orthopaedics and Trauma Surgery (main focus on trauma surgery), University Hospital of the RWTH Aachen, Aachen, Germany; Kaohsiung Chang Gung Memorial Hospital, Taiwan

## Abstract

**Introduction:**

Ischemia-reperfusion injury following cardiopulmonary resuscitation (CPR) is associated with a systemic inflammatory response, resulting in post-resuscitation disease. In the present study we investigated the response of the pleiotropic inflammatory cytokine macrophage migration inhibitory factor (MIF) to CPR in patients admitted to the hospital after out-of-hospital cardiac arrest (OHCA). To describe the magnitude of MIF release, we compared the blood levels from CPR patients with those obtained in healthy volunteers and with an aged- and gender-matched group of patients undergoing cardiac surgery with the use of extracorporeal circulation.

**Methods:**

Blood samples of 17 patients with return of spontaneous circulation (ROSC) after OHCA were obtained upon admission to the intensive care unit, and 6, 12, 24, 72 and 96 h later. Arrest and treatment related data were documented according to the Utstein style.

**Results:**

In patients after ROSC, MIF levels at admission (475.2±157.8 ng/ml) were significantly higher than in healthy volunteers (12.5±16.9 ng/ml, p<0.007) and in patients after cardiac surgery (78.2±41.6 ng/ml, p<0.007). Six hours after admission, MIF levels were decreased by more than 50% (150.5±127.2 ng/ml, p<0.007), but were not further reduced in the subsequent time course and remained significantly higher than the values observed during the ICU stay of cardiac surgical patients. In this small group of patients, MIF levels could not discriminate between survivors and non-survivors and were not affected by treatment with mild therapeutic hypothermia.

**Conclusion:**

MIF shows a rapid and pronounced increase following CPR, hence allowing a very early assessment of the inflammatory response. Further studies are warranted in larger patient groups to determine the prognostic significance of MIF.

**Trial Registration:**

ClinicalTrials.gov NCT01412619

## Introduction

Although the number of patients successfully resuscitated from cardiac arrest (CA) is increasing, the mortality rate in the post-resuscitation period is due to the severity of neurological and myocardial dysfunction still dramatically high [1]. Following cardiopulmonary resuscitation (CPR), complex changes including a systemic inflammatory response [2], [3], myocardial dysfunction [4]–[6], endothelial activation [7], alterations of the coagulation system [5], [8], adrenal insufficiency [9], hyperglycemia [10] and arterial hypotension [4], [11] are frequently observed. Although some of these features have been described already several decades ago [12], the importance of the post-cardiac arrest syndrome has only been recently underscored and highlighted in the latest CPR guidelines [13]. Interestingly, many of the features, which characterize the post-cardiac arrest syndrome are also frequently observed in patients with severe sepsis or septic shock, which has led to the synonym “sepsis-like syndrome" [14].

Macrophage migration inhibitory factor (MIF) is a pleiotropic inflammatory cytokine with chemokine-like functions, which is rapidly released from pre-formed cytoplasmic pools of several cell types (including monocytes/macrophages, B and T cells, endothelial and epithelial cells and cardiomyocytes), in response to various noxious stimuli such as infection, inflammation or hypoxia [15], [16]. MIF plays a pivotal role in the control of the acute immune response [17], [18], [19] and mediates the pathogenesis of acute and chronic inflammatory conditions, including rheumatoid arthritis, septic shock, acute respiratory distress syndrome and atherosclerosis by promoting and amplifying monocyte and macrophage survival, MAPK signalling and/ or cytokine release [15], [18], [20]. In sepsis, inhibition of the MIF pro-inflammatory activity has previously proven beneficial in numerous animal models of endotoxemia, and gram-negative and gram-positive septic shock [21]–[23]. In addition, recent studies observed a strong association between poor outcome and high levels of MIF in patients with severe systemic inflammation, organ failure and/or acute respiratory distress syndrome [24], [25], [26].

In an apparent contrast, MIF has been demonstrated to offer protection from I/R-injury by activating adenosine monophosphate-activated protein kinase (AMPK) and inhibiting c-Jun N-terminal kinase (JNK)-induced apoptosis of cardiomyocytes [27], [28].

Given the central involvement of MIF in immunological processes linked to I/R-injury, we hypothesized that MIF levels may exhibit an early increase in patients successfully resuscitated from OHCA.

We therefore investigated MIF serum levels in patients after out-of-hospital cardiac arrest (OHCA). To assess the magnitude of MIF release, we compared MIF serum levels of post cardiac arrest patients with those obtained in healthy volunteers and with an aged- and gender-matched group of patients undergoing cardiac surgery with the use of extracorporeal circulation and cardioplegic arrest.

## Materials and Methods

### Patients and study design

After approval of the study by the local institutional review board committee Aachen, 17 patients with cardiac arrest of non-traumatic origin and 17 gender- and aged-matched patients undergoing cardiac surgery with extracorporeal circulation and cardioplegic arest were consecutively enrolled in this study. The study was registered at ClinicalTrials.gov (NCT number: NCT01412619) and the protocol for this trial and supporting CONSORT checklist are available as supporting information; see Checklist S1 and Protocol S1. The included patients after OHCA represent a random sample of a larger group of patients in which the influence of MTH on S-100 values after OHCA and predictive power after OHCA has been recently studied and published [29]. The study complied with the Declaration of Helsinki.

Exclusion criteria were age less than 18 years, severe pre-existing conditions including sepsis, stroke, previous CPR and cancer. Cardiac arrest was defined as the absence of respiration, palpable pulse and responsiveness to stimuli. CPR was performed in accordance to the European Resuscitation Council's (ERC) guidelines 2000, which were gradually replaced during the study period by the updated guidelines of 2005 [30]. After recovery of blood pressure and pulse for more than 1 hour after admission to the hospital, CPR was considered as “successful" and patients were included in this study.

### Treatment of patients

After completion of CPR and admission to the hospital, all patients were transferred to the intensive care unit (ICU) and received standardised intensive care treatment including mechanical ventilation, fluid substitution, tight glucose control, sepsis and vasopressor treatment. Additional interventions (e.g., heart catheterisation) were carried out if appropriate. The initiation of mild therapeutic hypothermia (MTH) was left at the discretion of the attending physicians since not being a standard recommendation in the earlier ERC guidelines. MTH was induced using ice bags and infusion of cold fluids.

Tracheal extubation was performed when standard extubation criteria were fulfilled. Patients were discharged from the ICU after fulfilment of standardized clinical discharge criteria.

### Data collection

Baseline characteristics regarding demographic and CPR related parameters as well as clinical data were collected immediately after hospital admission in the emergency department (baseline) and 6, 12, 24, 72 and 120 hours after admission using a web-based data entry system complying with the Utstein-Style, initiated by the German Society of Anaesthesia and Intensive Care Medicine as part of a quality assurance system [31].

A standardized neurological assessment was performed by an independent physician, using the cerebral performance categories (CPC) after 14 days. CPC 1 and 2 were considered as favourable neurological outcome, whereas CPC 3 to 5 labelled adverse outcome [32].

### Laboratory Tests

Serum samples for the determination of C-reactive protein (CRP), procalcitonin (PCT), tumor necrosis factor alpha (TNF-α) and interleukin 6 (IL-6) and MIF were taken from the supernatant of blood collected for routine laboratory analyses at the predefined time points. The inflammatory cytokines IL-6, TNF-α and the biomarkers CRP, PCT were quantified using commercially available automated systems (LIAison, DiaSorin, Dietzenbach, Germany, and KRYPTOR, Brahms AG, Hennigsdorf, Berlin, Germany). The serum levels of MIF were determined using an enzyme-linked immunosorbent assay (ELISA) as previously described [15]. MIF levels of OHCA patients were compared with MIF values obtained from 30 healthy volunteers [33] and from 17 patients undergoing elective cardiac surgery at our hospital. After approval by the institutional review board and obtainment of written informed consent, the latter were identified as matched pairs using the variables gender and age. Cardiac surgery was performed with the use of extracorporeal circulation and cardioplegic arrest according to our institutional routine as described previously [34]. Cardiopulmonary bypass (CPB) was performed in moderate hypothermia (28°C–32°C) on a conventional CPB circuit with cardiac arrest induced by the antegrade infusion of cold crystalloid cardioplegic solution.

### Statistical Analysis

All data were statistically analysed using a commercially available software package (SPSS 17.0 (SPSS inc., Chicago, IL, USA). All data are expressed as mean ± SD.

As primary endpoint, we studied the time course of MIF serum levels after successful resuscitation.

All data were checked for normal distribution using the Shapiro-Wilk-test. Differences between groups were compared using a repeated measurement analysis of variance to take into account the correlated observations within the groups. As fixed effects, we included the within-factor time, the grouping factor pre-study treatment (patients after ROSC vs. patients after cardiac surgery), and the interaction effect (group * time). The time-course of the various biomarkers within the OHCA-group was analysed using one-way-ANOVA. In case of significant results, post-hoc testing was performed using the Student's t-test with Bonferroni-adjustment for multiple measurements. The significance level of the fixed-effects results was adjusted for multiple hypotheses (i.e., for the number of all biomarkers (n=7) tested in the present investigation and in the study from which the present patients represent a random sample, see above). Hence, a *p*<0.007 was considered to indicate statistical significance.

## Results

Patients' characteristics and cardiac arrest-related data about treatment and outcome are presented in Table 1.

**Table 1:** Baseline characteristics for patients after ROSC.

		Patients after ROSC(n=17)		
**Biometric/Demographic Data**				
Age (years)	mean ± SD	70	±	12
	median [IQR]	74		[61–79]
Sex, male	n (%)	10		(59)
**Arrest related data**				
VF as initial rhythm	n (%)	8		(47)
Asystole as initial rhythm	n (%)	6		(35)
Cardiac origin	n (%)	13		(76)
Call response interval (mm:ss)	mean ± SD	03:36	±	01:32
	median [IQR]	04:00		[03:00–05:00]
Number of shocks	n [IQR]	1		[1–3]
Arrest witnessed	n (%)	12		(71)
Location at public place	n (%)	5		(29)
Location at home	n (%)	11		(65)
**Treatment**				
Early defibrillation	n (%)	1		(6)
Bystander CPR	n (%)	3		(18)
Mild therapeutic hypothermia	n (%)	9		(53)
**Outcome**				
Mean survival (days)	median [IQR]	81		[52–192]
Survivors	n (%)	8		(47)
Good neurological recovery	n (%)	5		(29)
CPC (n)	median [IQR]	5		[2–5]

n=absolute number; (%)=percentage of the whole; IQR=interquartile range; SD=standard deviation.

VF=ventricular fibrillation; CPR=cardiopulmonary resuscitation; CPC=cerebral performance categories.

**Figure 1:**
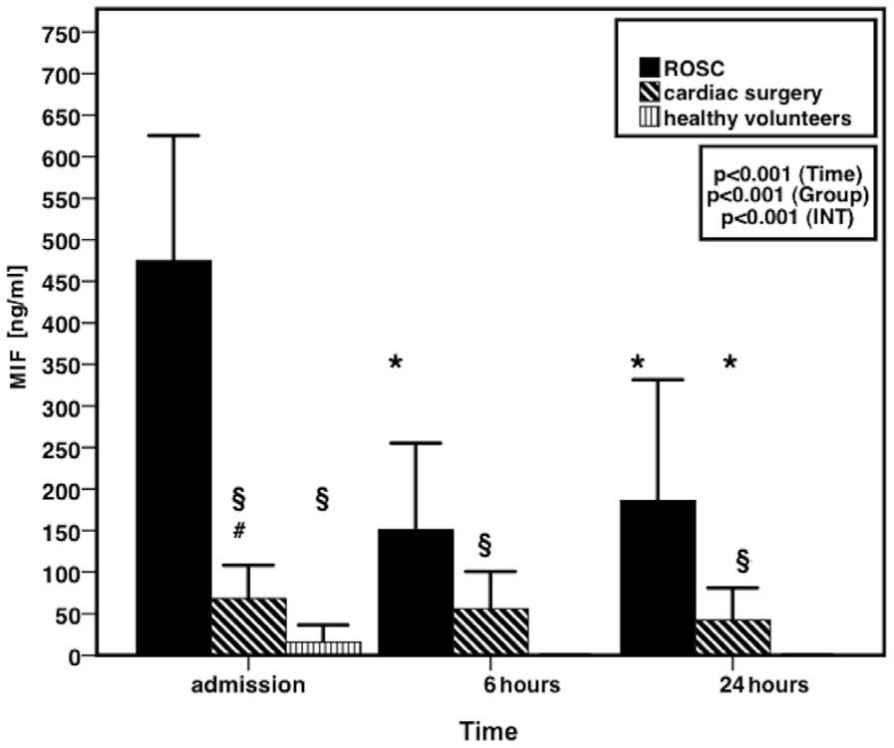
Comparison of MIF levels in patients after ROSC with those obtained in patients after cardiac surgery. Values are represented as mean ±*SD* at predefined timepoints after succesful CPR. MIF levels of healthy volunteers are additionally depicted at the time point “admission". * p<0.007 vs. baseline. § p<0.007 vs patients after ROSC. # p<0.007 vs. group of healthy volunteers.

The characteristics of the matched cardiac surgical patients are depicted in Table 2. In patients after ROSC, MIF levels at admission were significantly higher than in healthy volunteers [age 29±8 years; male=18 (60%)] and in patients after cardiac surgery (Fig. 1). There was a significant correlation between the call-response-interval and MIF levels upon admission (r=0.652; p=0.041). Six hours after admission, MIF levels decreased by more than 50%, but were not further reduced during the following time course and remained significantly higher than the values observed during the ICU stay of cardiac surgical patients (Fig. 1).

**Table 2:** Baseline characteristics for patients after cardiac surgery.

		Patients after cardiac surgery(n=17)		
**Biometric/Demographic Data**				
Age (years)	mean ± SD	70	±	11
	median [IQR]	74		[61–79]
Sex, male	n (%)	10		(59)
Euroscore	median [IQR]	5		[2–7]
**Type of surgery**				
Isolated CABG	n (%)	6		(35)
Isolated valvular surgery	n (%)	3		(18)
Aortic surgery	n (%)	1		(6)
Combined procedure	n (%)	7		(41)
Duration of surgery (min)	median [IQR]	188		[156–254]
Ischemia time (min)	mean ± SD	61.8	±	47.0
	median [IQR]	61.0		[41–83]
CPB Time (min)	mean ± SD	108.3	±	71.0
	median [IQR]	87	±	[70–125]

n=absolute number; (%)=percentage of the whole; IQR=interquartile range; SD=standard deviation.

CABG=coronary artery bypass graft; CPB=cardiopulmonary bypass.

**Figure 2:**
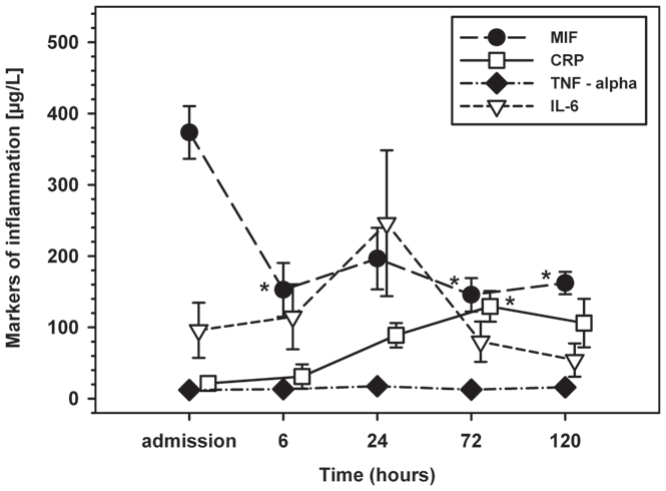
Serum levels of MIF, TNF-α, IL-6 and CRP after admission to the ICU and 6, 24, 72 and 120 hours later. * p<0.007 vs. baseline.

Of note, MIF levels peaked earlier than those of other inflammatory cytokines and sepsis markers. In comparison to CRP, TNF-α and IL-6, MIF showed its highest peak already at admission and was therefore the first cytokine to peak (Fig. 2). MIF then showed a second peak 24 h after admission to the hospital, which was paralleled by an IL-6 peak. In contrast, CRP levels were constantly increasing after admission. In order to compare the time course of inflammatory reaction in CPR patients with that after cardiac surgery, we additionally assessed the postoperative PCT release in the control group consisting of cardiac surgical patients. In both groups, PCT showed a similar time course with an increase reaching its maximum at 24–48 hours after admission to the ICU (Table 3).

**Table 3:** Comparison of time course of inflammatory reaction in patients after OHCA and after cardiac surgery as assessed by the serum levels of procalcitonin (µg/l).

		Admission				24 hours				48 hours				72 hours				120 hours			
**OHCA**	mean ± SD	0.21	±	0.35	n=17	7.16	±	11.61	n=13	n.a.				1.89	±	3.18	n=11	0.44	±	0.63	n=7
	median [IQR]	0.08		[0.05 –	0.13]	0.66		[0.19–	3.82]	n.a.				0.4		[0.19–	1.2]	0.18		[0.09 –	0.36]
**Cardiac surgery**	mean ± SD	0.58	±	0.74	n=17	4.95	±	13.24	n=17	11.63	±	16.48	n=6	n.a.				n.a.			
	median [IQR]	0.4		[0.10 –	0.50]	1.30		[0.40–	2.30]	5.45		[1.0 –	10.3]	n.a.				n.a.			

IQR=interquartile range [IQR].

n.a.=not available.

Results of the overall RMANOVA: p=0.71 (group); p=0.02 (time); p=0.59 (interaction).

**Figure 3:**
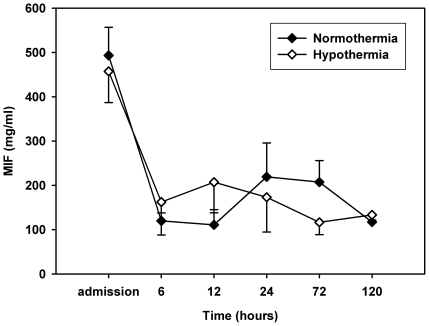
MIF serum levels in patients who recieved MTH in comparison with normothermically treated patients. Values are represented as mean ±*SD* at predefined timepoints after succesful CPR.

**Figure 4:**
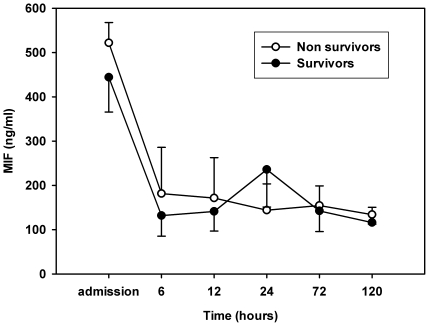
MIF serum levels in survivors and non-survivors. Values are represented as mean ±*SD* at predefined timepoints after succesful CPR.

The treatment with mild therapeutic hypothermia did not affect MIF serum levels (Fig. 3). In this small group of patients, MIF levels were neither able to discriminate between patients with good and bad neurological outcome nor to distinguish between survivors and non-survivors (Fig. 4).

## Discussion

The results of our study show that patients after OHCA exhibit a remarkable increase in serum protein levels of the pleiotropic cytokine MIF, which is positively correlated with the call-response-interval. MIF levels peak earlier and show a distinct dose/time response compared to other inflammatory and sepsis markers.

Successful CPR in patients after OHCA regularly elicits an ischemia/reperfusion-related release of proinflammatory cytokines and hence provokes a systemic inflammatory response. MIF is known to be expressed upstream during the inflammatory cascade [24]. During the pathogenesis of septic diseases, MIF plays a key role in up-regulating Toll-like receptor-4 (TLR-4), overriding glucocorticoid activity and stimulating the release of inflammatory cytokines such as TNF-, IL-1, IL-6 and IL-8 [17], [20], [24], [35]–[37]. In fact, we observed MIF to peak in advance of other key inflammatory cytokines.

MIF is an important mediator of I/R-injury being rapidly released in response to ischemia. Interestingly, our data indicate that the magnitude of MIF release is apparently related to the duration and the extent of ischemia. In our patients having suffered from total circulatory arrest and hence whole-body ischemia, MIF levels were correlated with call-response intervals that can be considered as a surrogate for ischemia time. Moreover, MIF levels at admission were increased nearly 30-fold in comparison with healthy volunteers (not having undergone ischemia), but “only" 9-fold in comparison with gender- and age-matched patients admitted to the ICU after cardiac surgery involving cardioplegic arrest and hence (in contrast to the OHCA-patients suffering from total circulatory arrest) “only" myocardial ischemia.

Recent evidence indicates that MIF can have both exacerbating (‘inflammatory’) and protective (‘anti-inflammatory’) effects in disease [18], [15], [38], but the mechanisms and details of the molecular decisions are only partly understood. In the context of the current study it is noteworthy of mentioning that secreted myocardial MIF is able to reduce I/R injury by suppressing apoptotic pathways and by activating the AMPK pathway through the MIF receptor CD74 [27], [28], [35].

On the other hand, studies in patients with severe systemic inflammation, organ failure and/or acute respiratory distress syndrome observed a strong association between poor outcome and high levels of MIF, which could be explained by its pro-inflammatory activity [17], [37]. Likewise, studies in patients after cardiac surgery observed an association between MIF-release and the occurrence of organ dysfunction during the postoperative course [25], [39]. These at first-sight contradictory observations may be readily explained by the complexity of the MIF/MIF receptor system. MIF not only interacts with CD74, but also engages in high affinity interactions with the chemokine receptors CXCR2 and CXCR4 to drive inflammatory leukocyte recruitment processes [18], [26] and may mediate the inflammatory pathogenesis of experimental atherosclerosis [26]. Binding of MIF to the transmembrane protein CD74 induces its phosphorylation and the recruitment of CD44, that further activates Src family nonreceptor tyrosine kinases and leads to ERK1/2 phosphorylation [35]. In addition, MIF has intracellular activities [15] and most recently a MIF-like homolog, MIF-2 or DDT, has been identified to have similar but not identical effects in inflammatory disease [40]. In our limited patient trial circulating MIF levels did not allow to discriminate between survivors and non-survivors and were not able to identify patients with good neurological outcome. We could therefore not confirm that high levels of MIF are closely linked to the occurrence of adverse events. Likewise, previous studies also failed to show any differences in the plasma levels of PCT, IL-6 and CRP in survivors and non-survivors after OHCA [3].

Interestingly, MTH did not affect MIF plasma levels, while in previous studies, MTH was associated with a decrease of inflammatory cytokines [3]. This finding might be attributed to the fact that the MIF release primarily occurs in direct response to ischemia and hence prior to the initiation of MTH. In fact, the largest MIF-peak was observed at admission to the ICU.

We acknowledge that our study is subject to several limitations. The control-group consisting of healthy volunteers was not age-matched. However, it is unlikely that the elevation of MIF after OHCA is merely attributable to an age effect as MIF values after OHCA were significantly higher than those observed in an age-matched group after cardiac surgery. Second, the number of included patients is relatively small. Hence, the study lacks statistical power to conclusively elucidate the prognostic significance of MIF after OHCA. Our results should therefore be considered as purely hypothesis-generating. Further studies are warranted to identify the prognostic role of MIF in a greater patient population. Our group has recently reported the prognostic significance of PCT levels in patients suffering from cardiac arrest [2]. However, MIF determinations in CPR patients could have a major clinical advantage as MIF peaks in advance to other inflammatory parameters, and this already at admission to the hospital. It has to be established whether MIF-determinations could offer early diagnostic evaluation and stratification of these patients upon admission to the hospital/intensive care unit. Third, it would have been very interesting to include a further control group of patients undergoing cardiovascular surgery with circulatory arrest and hence transitory whole body ischemia. On the other hand, validity of this comparison could be questioned due to the fact that – unlike during cardiac arrest - circulatory arrest during cardiac surgery is usually performed under the protection of deep hypothermia.

In conclusion, patients with successful resuscitation after OHCA show a remarkable increase of MIF serum levels upon admission to the ICU. MIF is the first cytokine to peak after CPR. Further studies are warranted to assess the effects on organ dysfunction and prognostic role of this early MIF peak.

## Supporting Information

Protocol S1 Trial Protocol.
(DOC)

Checklist S1 CONSORT Checklist.
(DOC)
