# Supplementary material for: Blood Levels of Macrophage Migration Inhibitory Factor after Successful Resuscitation from Cardiac Arrest
Source: PLoS One. 2012 Apr 10;7(4):e33512. doi: 10.1371/journal.pone.0033512 (PMC3323606; doi:10.1371/journal.pone.0033512)
Supplement: Protocol S1 — Trial Protocol. (DOC) [file pone.0033512.s001.doc]

**Protocol S1: Study protocol**

**Patients:**

The included patients (n=17) of the present trail represent a random sample of a larger group of patients in which the influence of mild therapeutic hypothermia on S-100 values after out of hospital cardiac arrest (OHCA) and the predictive power have recently been investigated and published [1]. Serum levels of macrophage migration inhibitory factor (MIF) were compared with those obtained from 17 gender- and aged-matched patients undergoing cardiac surgery with the use of extracorporeal circulation that were consecutively enrolled after obtainment of written informed consent.

To describe the magnitude MIF release, serum values were compared with MIF values obtained from 30 healthy volunteers [2].

**Exclusion criteria:**

Exclusion criteria were age less than 18 years, severe pre-existing conditions including sepsis, stroke, previous CPR and cancer.

**Definition and performance of cardiopulmonary resuscitation (CPR):**

Cardiac arrest was defined as the absence of respiration, palpable pulse and responsiveness to stimuli. CPR was performed in accordance to the European Resuscitation Council`s (ERC) guidelines 2000, which were gradually replaced during the study period by the updated guidelines of 2005 [3]. After recovery of blood pressure and pulse for more than 1 hour after admission to the hospital, CPR was considered as “successful” and patients were included in this study.

**Treatment of patients after successful CPR**

After completion of CPR and admission to the hospital, all patients were transferred to the intensive care unit (ICU) and received standardised intensive care treatment including mechanical ventilation, fluid substitution, tight glucose control, sepsis and vasopressor treatment. Additional interventions (e.g., heart catheterisation) were carried out if appropriate. The initiation of mild therapeutic hypothermia (MTH) was left at the discretion of the attending physicians since not being a standard recommendation in the earlier ERC guidelines. MTH was induced using ice bags and infusion of cold fluids.

Tracheal extubation was performed when standard extubation criteria were fulfilled. Patients were discharged from the ICU after fulfilment of standardized clinical discharge criteria.

**Data collection**

Baseline characteristics regarding demographic and CPR related parameters as well as clinical data were collected immediately after hospital admission in the emergency department (baseline) and 6, 12, 24, 72 and 120 hours after admission using a web-based data entry system complying with the Utstein-Style, initiated by the German Society of Anaesthesia and Intensive Care Medicine as part of a quality assurance system [4]. A standardized neurological assessment was performed by an independent physician, using the cerebral performance categories (CPC) after 14 days. CPC 1 and 2 were considered as favourable neurological outcome, whereas CPC 3 to 5 labelled adverse outcome [5].

**Laboratory Tests**

Serum samples for the determination of C-reactive protein (CRP), procalcitonin (PCT), tumor necrosis factor alpha (TNF-α) and interleukin 6 (IL-6) and MIF were taken from the supernatant of blood collected for routine laboratory analyses at the predefined time points. The inflammatory cytokines IL-6, TNF-α and the biomarkers CRP, PCT were quantified using commercially available automated systems (LIAison, DiaSorin, Dietzenbach, Germany, and KRYPTOR, Brahms AG, Hennigsdorf, Berlin, Germany).

**MIF ELISA**

The serum levels of MIF were determined according to our institutional standard using an enzyme-linked immunosorbent assay (ELISA) as previously described [6]. The human MIF ELISA was performed using capture antibody MAB289 and detection antibody BAF289 from R&D Systems (Wiesbaden, Germany).

**Cardiopulmonary bypass (CPB)**

CPB and cardioplegic arrest was performed according to our institutional routine as previously described [7] in moderate hypothermia (28°C–32°C) on a conventional CPB circuit with a pump flow of 2.2 l·min^-1^·m^-2^. Cardiac arrest was induced by the antegrade infusion of cold crystalloid cardioplegic solution. Prior to CPB, 300 U kg^-1^ heparin were administered to achieve an activated clotting time of > 400 sec. After weaning from CPB, heparin was antagonized with protamine at a ratio of 1:1.

**References:**

1. Derwall M, Stoppe C, Brücken D, Rossaint R, Fries M (2009) Changes in S-100 protein serum levels in survivors of out-of-hospital cardiac arrest treated with mild therapeutic hypothermia: a prospective, observational study. Crit Care 13:R58.

2. Grieb G, Simons D, Piatkowski A, Bernhagen J, Steffens G et al (2010) Macrophage migration inhibitory factor-A potential diagnostic tool in severe burn injuries? Burns 36:335-42.

3. Nolan JP, Deakin CD, Soar J, Bottiger BW, Smith G (2005) European Resuscitation Council guidelines for resuscitation 2005. Section 4. Adult advanced life support. Resuscitation 67 Suppl 1:S39-86.

4. Cummins RO, Chamberlain D, Hazinski MF, Nadkarni V, Kloeck W et al (1997) Recommended guidelines for reviewing, reporting, and conducting research on in- hospital resuscitation; The in-hospital ‘Utstein style’; a statement for healthcare professionals from the American Heart Association, the European Resuscitation Council, the Heart and Stroke Foundation of Canada, the Australian Resuscitation Council, and the Resuscitation Councils of South Africa. Circulation 95:2213-39.

5. Safar P (1981) Resuscitation after Brain Ischemia. In: Brain Failure and Resuscitation. Edited by Grenvik A, Safar P. New York: Churchill Livingstone 155-184.

6. Calandra T, Roger T (2003) Macrophage migration inhibitory factor: a regulator of innate immunity. Nat Rev Immunol 3: 791-800.

7. Stoppe C, Schälte G, Rossaint R, Coburn M, Graf B et al (2011) The intraoperative decrease of selenium is associated with the postoperative development of multiorgan dysfunction in cardiac surgical patients. Crit Care Med 39:1879-85.
